# Retraction: clinical and technical phosphoproteomic research

**DOI:** 10.1186/1477-5956-11-16

**Published:** 2013-04-23

**Authors:** Elena López, Isabel López, Antonio Ferreira, Julia Sequí

**Affiliations:** 1 Inflammatory core, Centro de Investigación i+12 del Hospital Universitario 12 de Octubre, Avda de Córdoba s/n, 28041, Madrid, Spain; 2 Hematology Department, Hospital Universitario 12 Octubre, Avda de Córdoba s/n, Madrid, 28041, Spain; 3 Immunology Department, Hospital Universitario La Paz, P° de la Castellana, Madrid, 261-28046, Spain; 4 Immunology Department, Hospital Carlos III, Sinesio Delgado, Madrid, 28029, Spain

## Retraction notice

This article [1] has been regretfully retracted by the Editors because of significant overlap with a figure and text from previously published articles [2-4]. We apologise to all affected parties for the inconvenience caused.
